# Critical roles of super-enhancers in the pathogenesis of autoimmune diseases

**DOI:** 10.1186/s41232-020-00124-9

**Published:** 2020-08-03

**Authors:** Kaoru Yamagata, Shingo Nakayamada, Yoshiya Tanaka

**Affiliations:** The First Department of Internal Medicine, School of Medicine, University of Occupational and Environmental Health, 1-1 Iseigaoka, Yahata-nishi-ku, Kitakyushu, 807-8555 Japan

**Keywords:** Super-enhancer (SE), Single nucleotide polymorphisms (SNPs), Genome-wide association study (GWAS), Enhancer RNA (eRNA), Autoimmune disease

## Abstract

The super-enhancer (SE) is a cluster of enhancers involved in cell differentiation via enhanced gene expression that determines cell identity. Meanwhile, genome-wide association studies (GWASs) have reported the presence of gene clusters containing single nucleotide polymorphisms (SNPs) susceptible to various diseases. According to cell types, these disease-susceptible SNPs are frequently detected in activated SE domains. However, the roles of SEs in the pathogenesis of various diseases remain unclear. This review first presents various functions of enhancer RNAs (eRNAs) transcribed from SEs. Next, it describes how SNPs and eRNAs are involved in the pathology of each autoimmune disease, with a focus on typical diseases such as rheumatoid arthritis, systemic lupus erythematosus, and multiple sclerosis. This review aims to describe the roles of SEs in the pathogenesis of autoimmune diseases through multiple interactions of these factors, as well as a future outlook on this issue.

## Background

Cells store genetic information in DNAs and synthesize RNAs by transcription. Furthermore, RNAs are translated into proteins, which perform specific biological functions. This process is referred to as the “central dogma,” proposed by Crick in 1958 [1]. In 1970, the splicing phenomenon was discovered, and the “one gene-multiple RNAs” hypothesis was proposed [2]. Moreover, scientists divided the transcription process into multiple stages, including initiation, elongation, and termination [3]. RNA polymerase II (RNAP II) is recognized as a core factor for the regulation of gene transcription. Gene expression is mediated by common transcription factors, promoters, enhancers, mediators, cohesin, insulators, and silencers [4]. Depending on circumstances, epigenetic mechanisms are involved. The aspects of transcriptional regulation have been expanded to include methylation of DNAs, phosphorylation of transcription factors, methylation and acetylation of histone, and modification of chromatin.

The enhancer is a short DNA sequence binding to proteins that activates gene transcription [5]. In 1981, the enhancer was first described as a repeated sequence of 72 base pairs in the simian virus 40 genome [6]. In 1983, mammal enhancers were discovered in mouse immunoglobulin heavy chain genes [7].

The concept of super-enhancers (SEs) has been developed to describe the clustering of enhancers. SEs, where mediators and cell-specific master transcription factors cluster, are considered to act as a switch to determine the cell fate and cell identity [8]. In addition, sequencing studies have proven that the SE domain is located in a topologically associating domain, which is a chromatin interacting region, where monomethylated histone 3 lysine 4 (H3K4me1), acetylated histone 3 lysine 27 (H3K27ac), and protein 300 (p300) are highly localized. The SE domain is characterized by a large DNA size, localization of many transcription factors, a high level of expression of enhancer RNAs (eRNAs: non-coding RNAs expressed from enhancers), and high transcriptional activity. Owing to these findings, SEs differ from typical enhancers [9]. It is considered that there are multiple promoters targeted during loop formation by SEs, while expressed target genes also vary [10].

In recent years, many regulatory factors involved in the regulation of SEs have been reported. Yin-Yang 1 (YY-1) is attracting attention as a factor structurally mediating DNA looping. YY-1 is considered to activate SEs throughout the genome [11]. On the other hand, SEs regulate the expression of organ-specific genes. In the brain, promoters and enhancers do not interact upstream of the special AT-rich sequence binding protein 1 gene, *Satb1*, and the expression of the *Satb1* is inhibited. On the other hand, SATB1 is highly expressed in the thymus, where the promoter and the enhancer interact [12]. A recent report has revealed that SATB1 induces the expression of the forkhead box protein 3 gene, *Foxp3*, via activation of SEs followed by differentiation of thymic regulatory T (Treg) cells [13]. The importance of the CCCTC-binding factor (CTCF), an insulator protein, has been highlighted. It is colocalized with cohesin on chromosomes and mediates chromatin-to-chromatin interaction [14]. Mediator of RNA polymerase II transcription subunit 1 (MED1), a transcriptional coactivator, plays an important role in the interaction between SEs and promoters. Reportedly, bromodomain-containing protein 4 (BRD4), another transcriptional coactivator, is colocalized with MED1. BRD4, which binds to acetylated histone, is important for the maintenance of H3K27ac, a marker for SEs. Furthermore, because BRD4 interacts with RNAP II, BRD4 is considered to be important for RNA transcription, especially the expression of eRNAs [15, 16]. Various eRNAs have been detected in SE domains; thus, eRNAs are reportedly involved in the regulation of SE activation [17].

In this review, we first present the various functions of eRNAs. Then, we describe the important roles of SEs in various autoimmune diseases from the perspectives of eRNAs and single nucleotide polymorphisms (SNPs). Furthermore, we discuss the future outlook for SE studies based on the pathogenesis of autoimmune diseases.

### Functions of eRNAs transcribed from SEs

eRNAs are transcribed from DNA sequences of enhancer regions, and confers average length of 350 nucleotides [18]. eRNAs are classified into two types according to length, transcriptional directionality, and polyadenylated state: 1D eRNAs and 2D eRNAs. Unidirectional transcripts generate long (over 4 kb) and polyadenylated eRNAs which are referred to as 1D eRNAs [19]. In contrast, bidirectional transcripts generate short (0.5–2 kb) non-adenylated eRNAs which are referred to as 2D eRNAs [20]. Most eRNAs expressed in human cell types are classified as 2D eRNAs.

The amount of detected eRNAs is 24.3 times greater in SEs than in typical enhancers [21]. Notably, in the case of macrophages, eRNAs are expressed in almost all SEs [22]. Thus, eRNAs can be regarded as a marker for SEs. As for biological functions, eRNAs are associated with (1) recruitment of transcription factors, (2) looping between SEs and promoters, (3) chromatin remodeling, (4) activation of RNAP II, (5) acetylation of histone, and (6) liquid phase separation, implying that eRNAs regulate gene expression.

YY-1 binds to not only enhancers but also eRNAs, stabilizing the DNA binding capacity [23]. As YY-1 binds to not only SEs but also promoters, eRNAs facilitate looping between SEs and promoters [24]. Meanwhile, nuclear factor kappa B (NF-kappa B) binding to the *interferon-gamma* (*IFN-gamma*) locus binds to eRNAs for *IFN-gamma* in immune cells such as naïve or memory T cells. When chromatin was treated with ribonuclease, the levels of NF-kappa B binding to the *IFN-gamma* locus decreased [25]. This result suggests that *IFN-gamma* eRNAs as a scaffold maintain the binding of NF-kappa B to the *IFN-gamma* locus.

**Fig. 1:**
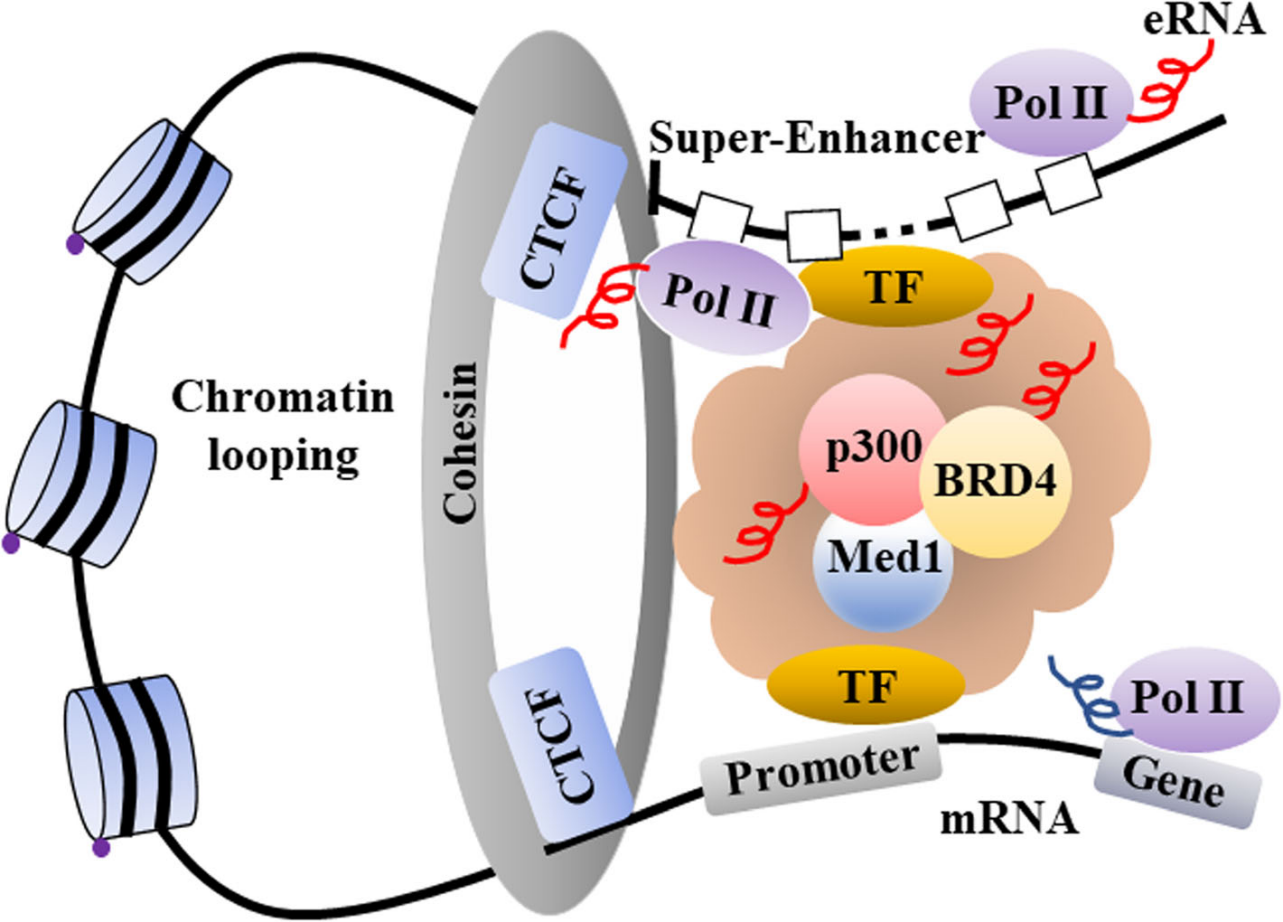
Schematic diagram of super-enhancer. SE is a cluster of enhancer that determines cell identity. Transcription factors (TFs) specific to cell types provide a platform for p300, which is recruited to SE domain, extensively mark H3K27me3. Protein complex includes cohesin, CTCF, Med1, and BRD4, and forms loop between SE and promoter, and then induces a powerful transcription of target gene. SE super-enhancer, eRNA enhancer RNA, CTCF CCCTC-binding factor, TF transcriptional factor, Med1 mediator of RNA polymerase II transcription subunit 1, BRD4 bromodomain-containing protein 4, RNAP II RNA polymerase II

As described above, looping between SEs and promoters is partially regulated by mediator complexes and cohesin complexes [26] (Fig. 1). RNA immunoprecipitation assay showed that eRNA interacted with RAD21 cohesin complex component (RAD21) and structural maintenance of chromosomes 3 (SMC3), the subunits of cohesin complex [27]. It has been reported that eRNA may interact with mediators like MED1 and MED17 in human embryonic kidney cells 293 (HEK293T) cells [28]. The knocking down of eRNAs reduced the loop formation rate [29–31]. Consequently, the recruitment of MED1, p300, and cohesin was also inhibited [29, 30]. For example, the knocking down of the eRNA for the growth-regulating estrogen receptor binding 1 gene, *GREB1*, reduced formation of loops between SEs and promoters; consequently, expression of the target gene *GREB1* decreased [32].

In skeletal muscle satellite cells, two types of eRNAs are expressed from SEs at the locus of the myogenic differentiation 1 gene, *MYOD1*. When the expression of these eRNAs was inhibited, the recruitment of RNAP II was inhibited at the *MYOD1* and *Myogenin* loci. Although the *Myogenin* locus is not sensitive to human deoxyribonuclease I (DNase I), chromatin remodeling creates a transcription-active state and sensitizes the locus to DNase I [33]. As the knocking down of eRNAs reduces the sensitivity to DNase I, chromatin remodeling may have been inhibited. Based on these findings, eRNAs are assumed to induce chromatin remodeling.

Negative elongation factors (NELFs) bind to RNAP II to inhibit transcription by RNAP II. The separation of NELFs from RNAP II is crucial for the synthesis of messenger RNAs (mRNAs). Inhibition of architectural eRNAs (*Arc* eRNAs) maintains the binding of NELFs to RNAP II [34]. This finding revealed a novel mechanism in which NELFs bind to eRNAs, are separated from RNAP II, and promote mRNA synthesis.

It is known that p300, which modifies H3K27ac, binds to cyclic adenosine monophosphate response element-binding protein (CBP), also a histone acetyltransferase (HAT). Bose et al. found eRNA species that bind to the HAT domain of CBP. Inhibition of eRNAs reduced H3K27ac of histone and inhibited the expression of target genes. This revealed a mechanism in which eRNAs induce the expression of target genes by enhancing the activity of HAT in CBP [35].

**Fig. 2:**
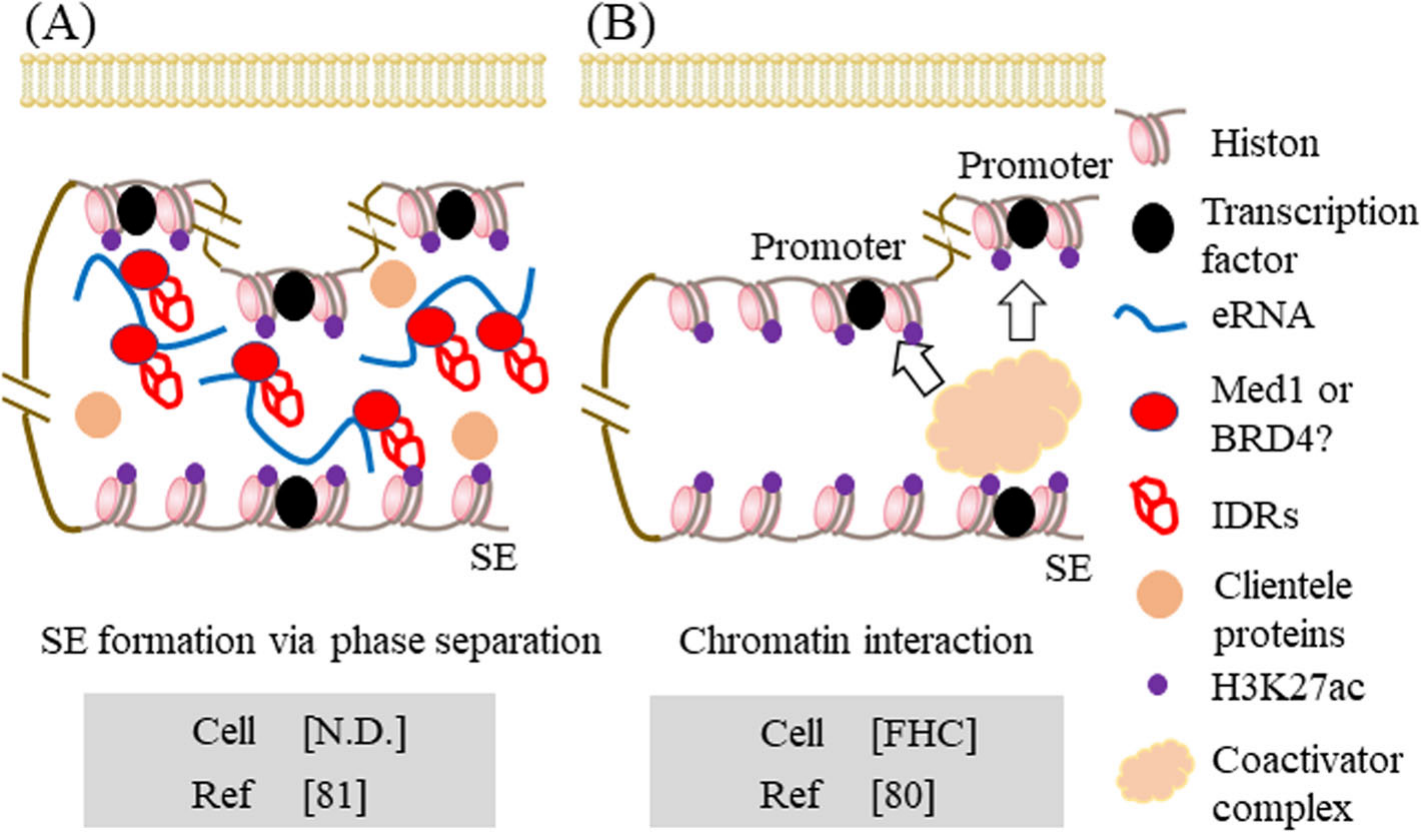
Novel mechanisms for activation and formation of SEs. **a** eRNAs bind to Med1, BRD4, and IDRs, and then form SEs via liquid phase separation. Disease-susceptible SNPs may affect phase-separated structure by altered structure of eRNAs. When SE dysregulation is caused, expression patterns of target genes may be altered. **b** SE alone forms loop with multiple promoters, and then regulates expression of target gene cluster. When disease-susceptible SNPs affect formation of chromatin loop, expression profile of target gene cluster may be altered. eRNA enhancer RNA, MED1 mediator of RNA polymerase II transcription subunit 1, BRD4 bromodomain-containing protein 4, IDRs intrinsically disordered regions, N.D. not determined, SNP single nucleotide polymorphism, SE super-enhancer, FHC fetal human cells

In the presence of abundant N6-methyladenosine (m^6^A), mRNAs induce the phase separation of cytoplasmic proteins. The m^6^A site functions as a binding platform for YT521-B homology domain family 2 (YTHDF2) and induces phase separation by using intrinsically disordered regions (IDRs) [36]. Importantly, this m^6^A modification is prominent in eRNAs [37]. The interaction of eRNAs with MED1, BRD4, and IDRs leads to the formation of SEs through phase separation [15] (Fig. 2a).

### Many SNPs susceptible to autoimmune diseases are located in SE domains.

There are more than 80 autoimmune diseases, and they affect 3 to 5% of the entire population in the USA [38, 39]. Human leukocyte antigen (HLA) class I is expressed in almost all cell types and presents peptide antigens on the cell surface. In contrast, HLA class II is expressed exclusively on dendritic cells and B cells, presenting antigens on the cell surface. SNPs in the *HLA class I/II* genes encoded on chromosome 6p21.3 are associated with various autoimmune diseases. Additionally, the presence of SNPs susceptible to various autoimmune diseases has been reported based on large-scale analysis of genome-wide association studies (GWASs) [40–50]. Meanwhile, Farh et al. focused on the involvement of various SEs and eRNAs in autoimmune diseases [51]. Subsequently, large-scale meta-analyses combining data on cell-specific SEs and disease-susceptible SNPs revealed more loci at risk for autoimmune diseases in immunocompetent cell-specific SEs than in protein-coding regions [41, 50] (Table 1). In T cells, because 1/3 of non-coding RNAs are transcribed from SEs, eRNAs may be involved in the immune response of T cells [41]. Furthermore, many gene clusters encoding cytokine receptors and cytokines in T cells have the SE structure [41]. Thus, hyperactivity or failure of each SE may lead to the pathogenesis of a set of autoimmune diseases [41, 52–58] (Table 2).

**Table 1:** Disease-susceptible SNPs in super-enhancers

Autoimmune diseases	Affected cell types	Disease-sensitive SNPs (*n*)	SNPs within SE (*n*)	Ref.
Rheumatoid arthritis	CD4^+^ T	101	27	[40, 41]
Systemic lupus erythematosus	N.D.	over 60	N.D.	[42, 43]
Multiple sclerosis	CD4^+^ T	87	36	[41, 44]
Systemic sclerosis	N.D.	27	N.D.	[45]
Graves’ disease	N.D.	101	N.D.	[46]
Behcet’s disease	N.D.	19	N.D.	[47]
Atopic dermatitis	N.D.	31	N.D.	[48]
Vitiligo	N.D.	over 30	N.D.	[49]
Inflammatory bowel disease	CD4^+^ T	216	91	[41, 50]

*SNPs* single nucleotide polymorphisms, *SE* super-enhancer, *N.D.* not determined

**Table 2:** Involvement of super-enhancer in autoimmune diseases

Autoimmune diseases	Affected cell types	SE/eRNA related to diseases	Genes regulated by SE/eRNA	Function	Ref.
Rheumatoid arthritis	CD4^+^T cells	BACH2 SE	*IFN-g*	Immune response	[41]
Juvenile idiopathic arthritis	CD4^+^ memory	CTLA4 SE	*CTLA4*	Preserve self-tolerance	[52]
	Effector T cells	CXCR4 SE	*CXCR4*	Cell infiltration	[52]
Systemic lupus erythematosus	Monocytes	Enhancer 1/2	*ADAMDEC1*	Maintenance of inflammation?	[53]
	PBMCs	PDCD1 SE	*PDCD1*	Preserve self-tolerance	[54]
Multiple sclerosis	THP-1 cells	VDR SE	*USP2*	Proinflammatory cytokine production	[55]
			*DENND6B*	Cytokine production during inflammation	[55]
Inflammatory bowel disease	CD14^+^ cells	IFNG-R-49	*N.D.*	*IL22* gene regulation	[56]
Vitiligo	Monocyte	HLA class II SE	*HLA-DR*, *-DQ*	N.D.	[57]
	PBMCs	HLA class II SE	*HLA-DR*, *-DQ*	IL-1b and IFN-g production	[57]
Autoimmune uveitis	Th1 cells	T-bet SE/eRNA	*IFNg etc.*	Cell invasion	[58]

*THP-1* human monocytic leukemia cell line, *PBMCs* peripheral blood mononuclear cells, *SE* super-enhancer, *eRNA* enhancer RNA, *BACH2* BTB and CNC homology 2, *VDR* vitamin D receptor, *CTLA* cytotoxic T-lymphocyte antigen, *CXCR* CXC chemokine receptor, *IFNG-R* interferon gamma-receptor, *HLA* human leukocyte antigen, *USP* ubiquitin-specific protease, *DENND6B* DENN domain-containing 6B, *ADAMDEC1* ADAM-like decysin-1, *N.D.* not determined

### Basic leucine zipper transcription factor 2 involved in the pathogenesis of rheumatoid arthritis is regulated by SEs.

Rheumatoid arthritis (RA) is a systemic autoimmune disease characterized by chronic synovial inflammation and progressive joint destruction [59–61]. In patients with RA, proinflammatory cytokines (e.g., tumor necrosis factor [TNF]-α and interleukin [IL]-6) and proteases (e.g., matrix metalloproteinase [MMP]-3) increase in the synovial fluid. Furthermore, lymphocytes infiltrate into the synovial membrane and play an important role in the pathogenesis of RA [62, 63]. Thus, TNF inhibitors, IL-6 receptor inhibitors, cytotoxic T-lymphocyte antigen 4 immunoglobulin (CTLA4-Ig), and small molecular weight compounds such as Janus kinase (JAK) inhibitors have been developed and are actively utilized in clinical practice [64–68]. Some patients achieve remission, whereas others show no remission or relapse. The existing drugs are not necessarily effective. On the other hand, based on previous genome analyses, the HLA-DRB1 SNP, known as a shared epitope, is known to be associated with high susceptibility to RA [69]. In addition, the results of GWAS analyses have revealed that 101 SNPs are associated with susceptibility to RA and cover half of the genomic variants underlying the susceptibility to RA [52, 70]. Furthermore, genomic information from GWAS and other studies provides a basis for precision medicine, which aims at providing optimal medical care [40]. Compared with typical enhancers, SEs harbor 3.2 times more SNPs susceptible to RA, suggesting that SNPs susceptible to RA are strongly associated with SE-mediated transcriptional regulation [46]. Moreover, 26% of SNPs (27/101) susceptible to RA are located within SEs activated by CD4^+^ T cells [41].

The basic leucine zipper transcription factor 2 (BACH2) protein is a key transcription factor for the maintenance of immune homeostasis by Treg cells [41]. In T cells, BACH2 inhibits the expression of genes encoding various cytokines, including IFN-gamma and cytokine receptors. Gene mutations at the *BACH2* locus are associated with RA. Knocking down of the *BACH2* gene induces the expression of various cytokines and their receptors [41, 71]. While the BACH2 protein negatively regulates the expression of eRNAs, the gene itself is uniquely regulated by SEs [41]. However, it is unclear which eRNA induces the expression of the *BACH2* gene or how eRNAs achieve this goal. Tofacitinib, which inhibits JAK 1/3, inhibits the expression of several genes susceptible to RA. Especially, it more dramatically inhibits the expression of genes regulated by SEs than the expression of genes not regulated by SEs [41]. This suggests a mechanism by which the JAK/signal transducer and activator of transcription (STAT) signals regulate the expression of genes susceptible to RA through SEs.

### Programmed death 1 and SEs’ importance for the pathogenesis of systemic lupus erythematosus

Systemic lupus erythematosus (SLE), which predominantly affects women of reproductive age, is an autoimmune disease that follows a chronic course or repeats relapse and remission. Heterogeneity is observed in some patients, and a complete cure is considered difficult to achieve with treatment [72]. Based on GWAS analysis, 60 disease-susceptible SNPs were discovered in European patients with SLE [42]. Additionally, nine new disease-susceptible loci were identified following analysis in Chinese patients [43]. However, the location and proportion of disease-susceptible loci in SE domains located throughout the genome are unknown. Disintegrin and metalloproteinase (ADAM)-like decysin-1 (ADAMDEC1) are important for proteolytic cleavage, but their detailed role remains unclear [53]. ADAMDEC1 is closely associated with ADAM28, which plays a role in the maintenance of the acute inflammatory process. Importantly, it has been reported that ADAMDEC1 is overexpressed in monocytes of patients with SLE and that its expression is induced by stimulation with proinflammatory cytokines. Furthermore, inflammatory stimulation results in the recruitment of NF-kappa B and p300 upstream of the *ADAMDEC1* gene. It was observed that eRNA-157 expressed from SEs bound to p300 in this process. In the absence of a bond between them, the induction of ADAMDEC1 expression was inhibited [53]. After enhancing the activity of p300 that marked with H3K27ac, eRNA-157 promoted induction of the *ADAMDEC1* gene expression via loop between promoter and SE. While eRNA-157 is a short non-coding RNA in a non-polyadenylated state generated by bidirectional transcript, *ADAMDEC1* mRNA is a long coding RNA in a polyadenylated state generated by unidirectional transcript. Whereas this eRNA-157 involves induction of *ADAMDEC1* mRNA, whether the latter regulates the former is unclear.

The programmed cell death 1 gene, *PDCD1*, encodes a programmed death 1 (PD-1) protein, which is important for immune checkpoint. *PDCD1*-knockout mice exhibit SLE-like pathology [73]. Moreover, SNPs of the *PDCD1* gene correlate with SLE [74]. Importantly, the *PDCD1* gene has the SE structure in CD4-naive T cells and is presumably regulated by SEs [75]. As the SE function may be impaired depending on the types of simulation, the pathogenesis of SLE may be promoted.

As described above, eRNAs and SEs that may be involved in the control of pathological conditions in patients with SLE have been identified. In the future, their functions will be elucidated at an individual level using mice and other disease models.

### Role of SEs in the pathogenesis of multiple sclerosis

Multiple sclerosis (MS) is a complex autoimmune disease caused by a combination of many risk factors, including genetic mutations and vitamin D deficiency [76]. SNPs susceptible to MS are observed at and around vitamin D receptor (VDR) binding sites [77]. Furthermore, such SNPs are frequently detected in activated SEs in CD4^+^ T cells (36/87) and monocytes [41, 55]. Lu et al. classified SEs bound to VDR, in other words, VDR SE (VSE), into 3 types: (1) VSE1 that is constantly bound to VDR and does not react to 1,25-dihydroxyvitamin D3 (1,25(OH)_2_D_3_); (2) VSE2 that is constantly bound to VDR and does not react to 1,25(OH)_2_D_3_, as well as that is bound by VDR depending on 1,25(OH)_2_D_3_; and (3) VSE3 that is bound by VDR depending on 1,25(OH)_2_D_3_. Several SNPs with susceptibility to MS were detected in VSE domains and were prominent especially in VSE3 domains [55]. Based on these findings, disease-susceptible SNPs within SEs are assumed to regulate SEs. However, it is unclear how the presence of SNPs susceptible to MS affects the expression of eRNAs via VDRs bound to vitamin D and chromatin interaction. This seems to be an issue for further investigation.

### In patients with vitiligo, risk SNPs in SEs increase the expression of major histocompatibility complex class II on peripheral blood mononuclear cells.

Vitiligo is an autoimmune disease characterized by white patches derived from progressive destruction of melanocytes by autoreactive T cells [78]. The pathogenesis of vitiligo is strongly associated with the major histocompatibility complex (MHC) class II region. Especially, it was associated with three risk SNPs (rs9271597, rs9271600, and rs9271601) located between the *HLA-DRB1* and *HLA-DQA1* genes. The retention of haplotypes of these risk SNPs was associated with a higher degree of localization of HLA-DR and HLA-DQ on the surfaces of monocytes than the absence of haplotypes [57]. When the peripheral blood mononuclear cells (PBMCs) of healthy individuals were stimulated with *Candida albicans*, which activates dectin and mannose receptor, and *Staphylococcus epidermidis*, which activates Toll-like receptor 2, production of IL-1 beta and IFN-gamma was enhanced in cells with haplotypes of high-risk SNPs in the MHC class II region, compared to cells with haplotypes of low-risk SNPs. This haplotype region has been identified as a SE in the B cell line GM12878. Additionally, this region has been reported as a transcription insulator [79]. Thus, the haplotypes may exhibit complex mechanisms of transcriptional activation and repression, depending on their combination [57]. Although these risk haplotypes are assumed to regulate SE activity, detailed elucidation of the transcriptional regulatory mechanism leading to increased expressed of MHC class II is crucial in future investigations.

### Roles of SEs in other autoimmune diseases

In the case of inflammatory bowel disease (IBD), approximately half of the risk SNPs (91/216) have been detected in the activated SE regions in CD4^+^ T cells [41]. However, there are many unknown aspects regarding the association between SNPs with susceptibility to many autoimmune diseases and SEs. Graves’ disease (GD) is associated with excessive humoral immunity due to the production of autoantibodies against the thyroid-stimulating hormone (TSH) receptor 1 [80]. GWAS has identified 101 SNPs susceptible to GD [46]. Meanwhile, atopic dermatitis (AD) chronically and repeatedly causes inflammatory allergic reactions, such as itching and flaking of skin. To date, large-scale GWAS analyses have identified 31 SNPs susceptible to AD [48] and 20 SNPs susceptible to Behcet’s disease [47]. In the future, it will be important to elucidate the involvement of SEs in a set of autoimmune diseases, including those described above [46, 49]. Whyte et al. reported that SEs are cis-regulatory elements and form loops with promoters [10]. In addition, SEs alone form loops with many promoters and consequently regulate the expression of many target gene clusters [81] (Fig. 2b). In normal colon cells (FHC), conversion from rs6854845-G to T using the clustered regulatory interspaced short palindromic repeat (CRISPR)/CRISPR-associated protein 9 (Cas9) system changed the expression patterns of many nearby genes (e.g., *CXCL8* [C-X-C motif chemokine ligand 8]) [82]. Thus, important themes for future investigation may be the identification of SNPs specific to each autoimmune disease by GWAS analysis, identification of SNPs important for chromatin interaction by linkage analysis, and measurement of actual interactions by chromosome conformation capture (3C) assay.

### Pathogenesis of autoimmune diseases by unique eRNA structures exhibiting single nucleotide mutations

Ren et al. revealed a region that expresses 23,878 eRNAs, covering 55.2 million base pairs (1.8%) in the human genome, in 50 human cell types, and tissues [82]. SNPs susceptible to autoimmune diseases clustered in regions expressing eRNAs in pathological cell types. Next, using the RNAsnp program, Ren et al. predicted the effect of SNPs with susceptibility to autoimmune diseases on the secondary structure of eRNAs [82]. They suggested that SNP rs6972403 correlated with the risk for AD and may substantially alter the structure of 3′ untranslated region of heat shock protein 83(Hsp 83 3′UTR) in lymphoid eRNA regions. The linkage disequilibrium between SNP3851228, which is associated with chronic bowel disease, and SNP11153299 or SNP2038013 was detected. The finding shows the two SNPs may alter the secondary structure of the TNF receptor-associated factor 3-interacting protein 2-antisense 1 (TRAF3IP2-AS1) in lymphoid eRNA regions. The altered secondary structure of eRNAs due to SNPs susceptible to autoimmune diseases, followed by the altered expression of target genes through chromatin regulation different from the original regulation may be involved in the pathogenesis of autoimmune diseases.

### Importance of SEs as candidate targets for RA treatment

Patients with RA have taken medications by so many different biologic agents as described in this review. However, their symptom does not necessarily respond to the medicated drugs and it is difficult at present to predict the response to various biologics. Studies on distribution of cell surface antigen have been conducted using PBMCs from RA patients to determine the effectiveness of the therapy and predict disease progression. As a practical example, researchers have attempted to predict therapeutic efficacy by grouping patients based upon the clinical parameters such as titers of autoantibody i.e., rheumatoid factor (RF), anti-cyclic citrullinated peptide antibody (ACPA), and levels of cytokines i.e., TNF-α and IL-6 etc. However, this classification does not necessarily lead to the best choice of medications for therapeutic efficacy. As another strategy, GWAS study found 101 SNPs with a susceptibility to RA, whose SNPs involve drug resistance in some cases [40]. Reportedly, SEs involve induction of various cytokines and their receptors. The SE activity may be affected by altered structure of eRNAs transcribed from SNPs locus frequently found within SEs [82]. Thus, a therapeutic strategy targeting eRNAs transcribed from SNPs locus susceptible to RA by locked nucleic acid (LNA) or antisense oligonucleotides (ASO) technique may have a good impact on therapeutic efficacy for RA.

### Future outlook for studies on SEs in autoimmune diseases

Estrogen, an important female hormone, binds to estrogen receptor α (ERα) to induce the expression of target genes and to play specific roles of estrogen. The following has been revealed through experimental studies: when the mammary gland cell line MCF-7 is stimulated with 17β-estradiol (E2), ERα binds to the response element on the genome, and p300, MED1, and BRD4 are recruited. H3K27ac is marked extensively depending on loci, and SEs are formed [83]. On the other hand, STAT proteins more preferentially bind to SEs than to typical enhancers in T helper (Th) 1 and Th2 cells. When STAT proteins are depleted, the recruitment of p300 to the loci decreases. Consequently, SEs are not formed to become active enhancers [41, 58]. These findings suggest a new concept of signal-induced formation of SEs (signal SEs) in addition to classic SEs conventionally formed in a signal-independent manner. As disease-susceptible SNPs may be located in signal SEs in addition to already-known disease-susceptible SNPs located in classic SEs, elucidation of the function of SNPs identified in GWAS is awaited.

#### Conclusion

SEs are activated according to cell types, and then play a role in forming cell identity. The autoimmune disease-susceptible SNPs identified in GWAS are frequently located in non-coding regions, as well as coding regions. Notably, many SNPs are located in SEs. Meanwhile, eRNAs regulate SE activity and are also associated with autoimmune diseases. Elucidation of the association between eRNAs and disease-susceptible SNPs is an important issue in autoimmune diseases. We anticipate that further studies on the mechanisms induced by abnormal SEs will elucidate the complex pathogenesis of each autoimmune disease in the future.

## Data Availability

Not applicable.

## Abbreviations

GWAS: Genome-wide association study
SNPs: Single nucleotide polymorphisms
SE: Super-enhancer
eRNA: Enhancer RNA
RAD21: RAD21 cohesin complex component
SMC3: Structural maintenance of chromosomes 3
HEK293: Human embryonic kidney cells 293T
IDRs: Intrinsically disordered regions
RA: Rheumatoid arthritis
SLE: Systemic lupus erythematosus
MS: Multiple sclerosis
IBD: Inflammatory bowel disease
GD: Graves’ disease
MED1: Mediator of RNA polymerase II transcription subunit 1
BRD4: Bromodomain-containing protein 4
RNAPII: RNA polymerase II
HLA: Human leukocyte antigen
BACH2: BTB and CNC homology 2
ADAMDEC1: ADAM-like decysin-1
VDR: Vitamin D receptor
VSE: Vitamin D receptor super-enhancer
JAK: Janus kinase
RF: Rheumatoid factor
ACPA: Anti-cyclic citrullinated peptide antibody
LNA: Locked nucleic acid
ASO: Antisense oligonucleotides
